# Population-Based Seroprevalence of Malaria in Hormozgan Province, Southeastern Iran: A Low Transmission Area

**DOI:** 10.1155/2015/174570

**Published:** 2015-10-12

**Authors:** Gholam Reza Hatam, Fatemeh Nejati, Tahereh Mohammadzadeh, Reza Shahriari Rad, Bahador Sarkari

**Affiliations:** ^1^Basic Sciences in Infectious Diseases Research Center, Shiraz University of Medical Sciences, Shiraz, Iran; ^2^Department of Parasitology and Mycology, School of Medicine, Baqiyatallah University of Medical Sciences, Tehran, Iran; ^3^Department of Parasitology and Mycology, School of Medicine, Shiraz University of Medical Sciences, Shiraz, Iran; ^4^Student Research Committee, Shiraz University of Medical Sciences, Shiraz, Iran

## Abstract

The seroepidemiological condition of malaria in three main districts of Hormozgan Province, a low transmission area in southeast of Iran, was investigated. *Methods.* Sera samples (803) were collected from healthy volunteers from the three main districts (Bandar Lengeh in the west, Bandar Abbas in the center, and Bandar Jask in the east) of Hormozgan Province. A questionnaire was used to record the sociodemographic features of the participants during sample collecting. An in-house ELISA test, using crude antigens obtained from cell culture of *Plasmodium falciparum*, was adapted and used to detect anti-malaria antibodies in the sera. *Results.* The overall seroprevalence of malaria was 8.7% (70 out of 803 samples). A significant correlation was found between seropositivity and place of residence, where the highest rate of seropositivity was seen in Bandar Lengeh (west of the province). The highest seroprevalence of malaria (13.2%) was seen in the age group of 11–20 years and also in low educated individuals. Correlation between seropositivity and gender, age, and educational levels of the participants was statistically significant (*P* < 0.05). *Conclusion. *Findings of this study indicate that the rate of seropositivity to malaria in this area is not high and this might be linked to the success of malaria control programs during the last decades in the region.

## 1. Background

Malaria is an infectious disease transmitted by the* Anopheles* mosquito and is caused by* Plasmodium* protozoan parasites. The disease is common in many tropical and subtropical areas of the world and is considered as an important life-threatening factor for people living in these regions. A total of 109 countries are considered as malaria-endemic regions and an annual count of 250 million cases of malaria occurs, in which close to 1 million cases are fatal [1].

Malaria has been considered as a threat in Iran with a high morbidity rate in the last decades [2–4]. Nowadays, the endemic regions of malaria in Iran are limited to the southern east part of the country, consisting of Sistan and Baluchestan, and southern Hormozgan and Kerman Provinces [5, 6]. The total cases of malaria in Iran progressively dropped from 11,460 cases in 2008 to 6,122 in 2009, 3,031 cases in 2010, and as low as 246 cases in 2014 (Iranian CDMC, unpublished data). Annual mortality of malaria in Iran is mostly related to the misdiagnosis of the disease in nonendemic regions of malaria in which physicians are not much familiar with the diagnosis and the treatment guidelines for malaria.

The predominant species of parasite in southern part of Iran is* P. vivax* and the main vector for the parasite is* Anopheles stephensi* [7].

The use of thin and thick smears for the diagnosis of malaria was conducted in 1900 and even nowadays it is considered as the gold standard in the diagnosis of malaria. Studies have shown that serological methods, for the diagnosis of malaria, can be considered as an effective tool in epidemiological studies and prevention programs of malaria, since these methods are able to detect previous exposure or contact with the disease.

Measuring the prevalence of anti-malaria antibodies (seroprevalence) is a valuable approach for defining the status of malaria transmission in a given area and also a useful method for malaria surveillance [8]. Moreover, seroprevalence is considered as a suitable and reliable tool for assessment of malaria transmission, especially in areas with low malaria transmission [9]. The current study aimed to assess the seroepidemiological condition of malaria in three main districts of Hormozgan Province, a low transmission area for malaria, in southeast of Iran.

As ELISA method consists of simplicity and low cost, compared with other serological methods, and is able to evaluate multiple samples simultaneously; therefore, in this study, ELISA method was used for the seroprevalence evaluation of malaria in a low transmission area of malaria in Iran.

## 2. Materials and Methods

### 2.1. Study Area

The current study was conducted in three main districts (Bandar Lengeh in the west, Bandar Abbas in the center, and Bandar Jask in the east) of Hormozgan Province, located in the southeast of Iran. Hormozgan is one of the 31 provinces in Iran and its provincial capital is Bandar Abbas. In 2011, Hormozgan Province had a population of 1,500,000 people. Hormozgan Province maintains a long warm (9 months) season and a short (three months) cool season. The province is one of the warm and dry regions of Iran, with warm and humid coastlines in summer, where the temperature climbs up to 52°C. The average annual temperature of the region is about 27°C. The province is considered as a malarious province in Iran.

### 2.2. Blood Samples

After getting approval from the Ethics Committee of Shiraz University of Medical Sciences, blood samples were taken from 803 healthy volunteers from three main districts of Hormozgan Province including Bandar Lengeh (171 samples), Bandar Abbas (457 samples), and Bandar Jask (153 samples). The samples were taken from the tip of the left second finger of each subject with the help of a disposable lancet. Three drops of blood were collected from each participant onto a filter paper (Whatman 3 MM), dried at room temperature, sealed in a plastic bag, and stored at −20°C until use. Cluster sampling, based on the population of each district, was used for sample collecting. These three districts are in National Malaria Control Program areas in Iran and names of the head of households were available in local health centers. Cases were randomly selected from these files and reached either in their house or in the health centers. Informed consent was obtained from the subjects before sampling.

### 2.3. *Plasmodium falciparum* Culture and Antigen Preparation

Trager and Jensen (1976) method was used for continuous culturing of* P. falciparum* with the help of two media, Albumax complete medium and alternative malaria complete medium (MCM) [10]. Strain of* P. falciparum* was kindly provided by Professor S. Zakeri (Pasteur Institute of Iran, Tehran). Malaria antigen was prepared from in vitro culture of* P. falciparum*. Briefly, HEPES was added to the contents of malaria culture flask, containing RPMI medium, and the content was centrifuged at 1800 g for 10 minutes. The pellet was diluted, 10 times, with the same medium and 2 mL of sample was added to 2.5 mL of cold Percoll (60%), in a 15 mL Falcon tube on ice. After centrifugation at 4°C for 15 minutes, the interphase layer containing parasites schizont was isolated. Then, 5% saponin in PBS was added to the sample and mixed thoroughly to eliminate the red blood cells. Sample was immediately centrifuged, 3200 g for 10 minutes, to remove the saponin. The obtained pellet was washed twice with PBS and kept at −20 until use.

### 2.4. Extraction of Sera from Whatman Papers

The blood spot on filter was punched with a paper punch and each punch was placed in a well of a U shaped ELISA microplate. Extraction of sera from filter papers was performed as previously described with some modification [11]. Briefly, PBS-T (PBST containing 0.05% Tween 20) was added to each well (125 *μ*L of PBS-T to each well, corresponding to 1/50 of serum dilution). The plate was placed on a shaker for at least 12 hours at 4°C and centrifuged with a plate centrifuge at 2200 g for 20 minutes. The supernatant from each well was removed and used for ELISA.

### 2.5. ELISA for Measuring Anti-Malaria Antibodies

ELISA microplate was sensitized with 5 *μ*g/mL of crude malaria antigen in coating buffer (0.05 M carbonate-bicarbonate buffer, pH 9.6). The plate was washed with PBS containing 0.05 Tween three times. Unbound sites were blocked, using skimmed milk (5%), and washed twice after 2 hours. Then, 100 *μ*L of serum containing solution, extracted from Whatman paper, was added to each well and incubated in room temperature for 1.5 hours followed by a wash-up as before. Horseradish peroxidase (HRPO) conjugated anti-human antibodies (Sigma) at 1/1000 dilution in PBST were applied to the plate and incubated at room temperature for 1 hour. Following washing, 100 *μ*L/well of chromogen/substrate (0.4 mg/mL OPD and 0.025% H_2_O_2_ in 0.1 M citrate buffer, pH 5) was added to the plates and the absorbance values of the wells, at 490 nm, were determined by a microplate reader, after 30 minutes. Negative and positive samples were included in each series. Negative controls sera were collected from people from nonmalarious area and those who had no history of malaria. Positive sera were selected from samples of our previous study [12]. Cut-off was determined, using mean of OD of negative samples plus 2 SD.

### 2.6. Statistical Analysis

Standard *χ* ^2^ test was used to assess the univariate correlation of demographic and malaria seropositivity.

## 3. Results

The mean age of participant was 27.4 years (range 1–74). Males constituted 58.3% of the subjects and 41.6 of them were females. The overall seroprevalence of malaria was 8.7% (70 out of 803 samples). The number of positive cases in Bandar Abbas was 15, in Bandar Lengeh it was 51 samples, and in Bandar Jask it was 2 cases. A significant correlation was found between seropositivity and place of residence. The highest seroprevalence of malaria (13.2%) was seen in the age group of 11–20 years. Seropositivity was higher in low educated individuals. Correlation between seropositivity and gender, age, and educational levels of the participants was significant.  Table 1 shows the sociodemographic characteristics of participants and relative seropositivity to malaria in this study.

## 4. Discussion

Despite the significant advances that have been made over the years to control malaria, the disease is still a major health problem in the world and about one hundred of the countries or regions of the world are among the malaria-endemic areas [2, 3]. In Iran, as a result of successful implementation of half a century of prevention and control programs, currently there is no malaria transmission in parts of the country and only in limited areas, mainly in the southeast of the country, the transfer of local malaria can be observed [3]. More than 91% of the malaria cases in Iran are from Sistan and Baluchestan, Hormozgan, and Kerman Provinces. The total malaria cases in Iran gradually dropped from 11,460 cases in 2008 to 6,122 in 2009, 3,031 cases in 2010, 519 cases in 2013, and as low as 246 cases in 2014 (Iranian CDMC, unpublished data).

Several studies pointed out that serological markers are valuable tools for evaluating and monitoring of malaria transmission [8, 9, 13]. In the current study, a serological test was applied to find out the rate of seroprevalence of malaria in an endemic area of malaria in southeast of Iran. So far, a few studies in Iran, in particular in the field of seroepidemiology of malaria, have been done in the malarious provinces [14–16]. In most of these studies, IFA technique is used. In the present study, because of its simplicity, low cost, and ability to perform several samples simultaneously, an in-house ELISA was used for the evaluation of seroprevalence of malaria in Hormozgan Province.

Moreover, in this study filter paper has been used for sample collecting which has several advantages. Perhaps the most important advantage is the elimination of blood sampling from the vessels, which often in infants and children have been the subject of debate. In epidemiological studies, obtaining blood samples from the vessels due to the required equipment is more expensive than obtaining blood samples from the capillaries. Another important advantage of this method is the ease of transfer and storage of samples before experiment. The method is less invasive and also reduces the risk of disease transmission.

In the present study, 8.7% of the participants had anti-*Plasmodium* antibodies. In a seroprevalence study conducted in Sistan and Baluchestan Province, anti-*P. vivax* and* P. falciparum* were detected in 31.6 and 5% of blood donors, respectively, by IFA [15]. It is worth mentioning that in our study crude antigen of* P. falciparum* was used in ELISA. This antigen contains pan malaria antigens which react with* P. vivax* and* P. falciparum* antigens. Therefore, antibodies against both species of malaria have been detected in the participants in the current study.

The seroprevalence of malaria in this study is lower compared with those studies conducted in other regions of the world. This might be related to the success of malaria control programs in the region and implies that eradication of the disease in the province in the coming years is feasible [17].

A very low level of seropositivity to malaria (about 1%) was reported in a previous study in another district (Bashagard) of the province. The main explanation of this low seropositivity is that the study has measured anti-malaria antibodies (IgG, IgG1, and IgG3) by ELISA, using either PvMSP-119 or PfMSP-119 antigens, instead of using a pan-malaria antigens [18]. Antibodies to these antigens have short half-life as documented before [19].

In this study, seropositivity and gender were significantly associated. In total, women had more antibodies compared to men. The reason for that is not clear since both males and females are equally exposed to* Anopheles* biting and the link between malaria susceptibility and gender has not been documented.

In the current study, the highest seropositive cases were from the age group of 11–20 years and the lowest in the age group of 1–10. The lower seroprevalence in children below ten years indicates less exposure of individuals and probably lack of local transmission of malaria. Higher rate of seropositivity in postsecondary educational level, in comparison with primary and secondary levels, may also be related to the age of the subjects rather than educational level.

Some of the seropositive cases in this study may have parasite, though at very low level, and may act as malaria reservoir, contributing to the persistence of malaria transmission. These seropositive asymptomatic cases may be a big challenge for malaria elimination program in any endemic area.

The study of Nateghpour et al. on 408 patients with suspected malaria symptoms microscopically detected* P. vivax* and* P. falciparum* in 17.9% and 1.7% of the cases, respectively. The serum samples have been seropositive with* P. vivax* and* P. falciparum* antigens in 54.2% and 32.1% of the samples, respectively [14]. The higher rate of seropositivity in Nateghpour study in comparison with the current study is because the subjects of their study have been malaria suspected individuals and, not surprisingly, the rate of seropositivity should be quite higher in these subjects [14].

Taken together, the findings of this study demonstrated a relatively low rate of seropositivity of malaria in Hormozgan Province, southeastern Iran.

## Figures and Tables

**Table 1:** Demographic characteristics of participant and relative seropositivity to malaria in Hormozgan Province, southern Iran.

Characteristics	Frequency (number)	Percent (%)	Positive for anti-malaria Ab.		*P* value
			Number	%	
*Gender*					
Male	469	58.3	15	3.2	<0.05
Female	334	41.6	55	16.47	
*Age group*					
1–10	54	6.75	1	1.43	<0.05
11–20	106	13.26	14	13.2	
21–30	369	46.18	37	9.75	
31–40	161	20.15	10	6.21	
41–50	70	8.76	2	2.86	
51 and higher	39	4.9	1	1.43	
*Residence*					
Bandar Abbas	457	58.51	15	3.28	<0.05
Bandar Lengeh	171	21.89	51	29.82	
Bandar Jask	153	19.59	2	1.31	
*Educational level*					
Uneducated	82	10.28	1	1.2	<0.05
Primary and secondary level	281	32.25	31	11.1	
Postsecondary level	253	31.74	32	12.64	
University level	123	15.4	6	4.87	
